# A Cross-Sectional Questionnaire Study of Tinnitus Awareness and Impact in a Population of Adult Cochlear Implant Users

**DOI:** 10.1097/AUD.0000000000000601

**Published:** 2018-12-28

**Authors:** Philip A. Gomersall, David M. Baguley, Robert P. Carlyon

**Affiliations:** 1 MRC Cognition & Brain Sciences Unit, University of Cambridge, United Kingdom; 2 Cambridge University Hospitals NHS Foundation Trust, Cambridge, United Kingdom; 3 National Institute for Health Research—Nottingham Hearing Biomedical Research Unit, Nottingham, United Kingdom; 4 Otology and Hearing, Division of Clinical Neuroscience, School of Medicine, University of Nottingham, Nottingham, United Kingdom.

**Keywords:** Cochlear implants, Tinnitus

## Abstract

**Objectives:**

The primary aim was to identify the proportion of individuals within the adult cochlear implant population who are aware of tinnitus and those who report a negative impact from this perception, using a bespoke questionnaire designed to limit bias. A secondary aim was to use qualitative analysis of open-text responses to identify themes linked to tinnitus perception in this population.

**Design:**

A cross-sectional questionnaire study of a large clinical population who received an implant from Cambridge University Hospitals, United Kingdom.

**Results:**

Seventy-five percent of respondents reported tinnitus awareness. When impact scores for six areas of difficulty were ranked, 13% of individuals ranked tinnitus their primary concern and nearly a third ranked tinnitus in the top two positions. Tinnitus impact was not found to reduce with duration since implantation. The most common open-text responses were linked to a general improvement postimplantation and acute tinnitus alleviation specific to times when the device was in use.

**Conclusions:**

Tinnitus is a problem for a significant proportion of individuals with a cochlear implant. Clinicians, scientists, and cochlear implant manufacturers should be aware that management of tinnitus may be a greater priority for an implantee than difficulties linked to speech perception. Where a positive effect of implantation was reported, there was greater evidence for masking of tinnitus via the implant rather than reversal of maladaptive plasticity.

## Abstract 2

Supplemental Digital Content is available in the text.

## INTRODUCTION

Tinnitus is the perception of sound without a corresponding external stimulus and is a common symptom that can be debilitating for some (Baguley et al. 2013). Injury to the peripheral auditory system is considered to be a trigger for tinnitus, and explanations for tinnitus onset linked with cochlear hearing loss indicate a complex, and as yet not comprehensively described, combination of (i) the disinhibition of auditory cortex neurons as a result of reduced cochlear nerve activity, resulting in increased firing rates, and (ii) increased temporal synchrony across populations of nerves that have reduced or absent corresponding peripheral input (Eggermont & Roberts 2004; Kaltenbach 2011; Saunders 2007). These alterations may form part of a plastic rearrangement of the auditory pathway at cortical and subcortical levels that results in a sustained perception of tinnitus (Noreña 2011). Given the above, it is unsurprising that reports of tinnitus within the population with severe-profound hearing loss, the majority of whom have longstanding acquired sensorineural hearing impairment, are high (67 to 90%); see Baguley and Atlas (2007) for a review.

### The Influence of Cochlear Implant on Tinnitus

The cochlear implant (CI) is the treatment of choice in the industrialized world for bilateral severe/profound sensorineural hearing loss. The potential benefits of use of the implant for tinnitus are increased activity in the peripheral auditory system (which may have a direct impact on the pathophysiological mechanisms of tinnitus) but also improved access to external sounds that will mask or distract the sufferer from the tinnitus perception (Quaranta et al. 2004). Improvements in psychological well-being and general quality of life arising as a result of better communication ability may also play a part in reducing the reported impact of tinnitus (Baguley & Atlas 2007). While no randomized controlled trial evidence exists, a systematic review of cohort and observational studies indicates a general trend in the literature of improvement in tinnitus in recipients of CI (Ramakers et al. 2015). In the studies reviewed, statistically significant reductions in tinnitus measures were documented pre- and postimplantation. We should not mistake these effects, seen at the group level, as a demonstration that all individuals with bothersome tinnitus undergo a detectable improvement in quality of life. Careful examination of these studies reveals that it is generally seen that tinnitus distress persists in a proportion of the population undergoing implantation.

Andersson et al. (2009) used a cross-sectional design to measure tinnitus impact in adults postimplantation. They found that 35% of individuals reported a moderate or severe handicap as measured by the Tinnitus Handicap Inventory (THI) (Newman et al. 1996) and that self-reported measures of tinnitus handicap correlated with self-reported anxiety, depression, and hearing difficulties. Comparison of THI scores with other tinnitus populations showed “broad congruence”. The authors concluded that a small but significant section of the adult implanted population suffer from significant tinnitus distress and recommend that clinical management options are considered that are tailored to the CI population.

There are a number of factors contributing to the variability in the estimates of individuals with CI who continue to perceive tinnitus postimplantation. Many studies included relatively small populations and often emphasized the identification of improvements in tinnitus handicap, rather than providing an estimate of the number of individuals who may continue to be in need of support by documenting tinnitus impact postimplantation. A range of outcome measures are used across studies, making direct comparisons difficult. The potential benefit of using validated questionnaires to assess tinnitus is less relevant to studies focusing on the CI population because the severity of hearing loss is worse and the duration of hearing loss is significantly longer in the implanted population than those used in validation studies for these self-report tinnitus tools. On this basis, we argue that the presently available tinnitus self-report measures are less appropriate for the CI population.

A further, more general, criticism of the widely used self-report tinnitus measures point is that the tinnitus percept can be modulated by attention (Knobel & Sanchez 2008) and reports of tinnitus may be different when that symptom is considered explicitly and in isolation, versus in the context of questions focusing on other areas of auditory disability. The multiple and specific questions about the impact of tinnitus in a questionnaire such as the THI or Tinnitus Functional Index (Meikle et al. 2012) may thus amplify the reported impact.

The approach in this study, novel for a study focusing on tinnitus perception, is to include a small number of questions focusing on other areas of potential difficulty for CI users alongside the tinnitus-specific question. Two particular benefits were anticipated:

1. Increased participation and minimized selection bias.

The simplicity and brevity of the questionnaire aimed to prevent barriers of language and literacy. The breadth of topics and inclusion of open text responses aimed to encourage responses from individuals without tinnitus and those who wished to elaborate on their quantitative responses.

2. An analysis of the relative judgments of tinnitus impact compared to these other areas of difficulty.

Relative judgments, compared to absolute judgments, are more robust to temporary external influences such as fluctuations in mood or a motivation to deliberately over- or under-emphasize a response. As an example, comparative analysis of the results may help negate a “baseline shift” across all responses that may occur if participants try to supply the answers they believe the experimenter is hoping to receive or to communicate a general sense of dis/satisfaction (Goffin & Olson 2011).

## MATERIALS AND METHODS

### Questionnaire Rationale and Design

To maximize participation and inclusivity, the questionnaire was simple to complete with only a small number of questions. Five areas (henceforth referred to as domains) that are reported as problematic in CI users were selected alongside tinnitus perception (speech perception in quiet, speech perception in noise, music perception, naturalness of speech, and naturalness of environmental sounds). We anticipated that a broad range of topics would increase the likelihood of positive outcomes (i.e., speech perception in quiet; Noble et al. 2008), as well as negative experiences (e.g., music perception; Limb & Roy 2014). Open-text responses encourage participation as subjects feel that they are able to better represent their personal experience/opinions (Elliott & Timulak 2005), with the potential to provide additional information about tinnitus experiences. Visual analog scales (VAS) were chosen for the closed responses. These have been shown to be reliable in the measurement of subjective phenomena, including tinnitus (Adamchic et al. 2012). Descriptive end points for the VAS were selected using guidance from the literature (Scott & Huskisson 1976) and were based on those of the Likert scales from the widely used hearing aid outcome measure, the Glasgow Hearing Aid Benefit Profile (Gatehouse 1999).

For each of the domains of potential difficulty, two VAS were created, each measuring 10 cm in length on the paper questionnaires. In each case, the first VAS refers to a quantification of the level of difficulty for that domain (i.e., “How difficult is it for you to follow a single person speaking in a quiet room”) and the second asks the participant to report the subsequent impact on their life (“How much does this worry/annoy/upset you”).

Subjects were given written instructions to “make a mark on the line to indicate your answer” (a copy of the questionnaire and instructions are included in Appendix A1, Supplemental Digital Content 1, http://links.lww.com/EANDH/A448).

### Population

Suitable adult individuals were identified from the database of the Emmeline Centre for Auditory Implants, Cambridge University Hospitals, United Kingdom, and 466 people were contacted.

Questionnaires were sent by post to all adult individuals documented to have undergone unilateral cochlear implantation from the Emmeline Centre for Auditory Implants in Cambridge, United Kingdom. Individuals receiving binaural implants were excluded due to the small number precluding a separate statistical analysis. Recipients were identified from the clinical database using the following exclusion criteria: age less than 18 years old on date of posting of questionnaires, any clinical note reporting significant visual impairment that might prevent the VAS from being completed, and clinical note of severe mental illness. Demographic information regarding gender, age, time since implantation, and the device used was extracted from the clinic database. One individual was known to have received an implant on a research basis for a sudden unilateral sensorineural hearing loss and debilitating tinnitus and was excluded from the study for this reason.

### Analysis of VAS Responses

VAS marks were measured to the nearest 1 mm. Where crosses were marked above or below the VAS, lines perpendicular to the VAS were drawn through the central part of the mark (most commonly a cross). In a small number of cases, marks were placed just outside the extremes of the VAS scale: these were recorded as being at the extreme value. VAS scores for the first section of each question are referred to as *impairment* scores, while scores for the second question are referred to as *impact* scores.

Ranking calculations were performed using a tied rank procedure. This assigns a shared ranking in the case of a tie (i.e., if the VAS *impact* scores for the 6 domains focused on were 5.4, 3.4, 3.4, 2.5, 1.3, and 1.2, then the corresponding ranks assigned would be 1, 2.5, 2.5, 4, 5, and 6). Half-scores were rounded up to the nearest integer for ease of presentation.

### Open Text Responses

To analyze the open-text responses, a simple content analysis was performed. This approach has been defined as “a research method that uses a set of procedures to make valid inferences from text.” We followed key steps outlined by two authors (Weber 1990; Stemler 2001) that involve the collation of the data, selection of a unit of analysis, and then application of coding rules to categorize the information contained in the response. Other qualitative approaches exist that may offer more discursive accounts of the open-text responses but that were not desired here. Our primary aim was to identify concepts or themes reported commonly, or in a pattern, across the respondents that may have supplemented, supported, or counteracted the findings of the closed-set responses. An inductive approach was used to develop categories from a subsample of the data, using two different individuals as “coders” to facilitate a check for reliability. The unit of analysis was selected to be the full passage written by the participant (commonly a single sentence or short paragraph of fewer than 30 words). A randomly selected sample of 37 of these responses (approximately one third of individuals who provided a tinnitus open-text response) was analyzed by one of the authors (P.G.), who suggested several categorizations (referred to as themes) with a number of subcategories assigned to each theme (Table 1, first column), to which codes were applied. The majority of themes were determined solely from the content provided, that is, all but one of the themes existed in the 37 samples initially examined (emergent coding), with one theme added on the basis of experience and theoretical knowledge about tinnitus. The definition of each theme identified was constructed so that subcategories were mutually exclusive where possible. Subcategories in each theme were generally defined on the basis of the content of the initial samples (referred to as emergent coding), but in addition further subcategories were added a priori. This was done to ensure that the theme was covered exhaustively in cases where this was suitable. For example, in the initial sample, it was noted that several reports indicated that the use of the implant lessened or completely suppressed tinnitus awareness. Consequently, the theme of “impact of specific use” was created, with two subcategories of “complete suppression,” and “improvement.” Two additional categories of “no impact” and “worsening of tinnitus” were added, in order that any response under the theme of “impact of specific use” (positive or negative) could be appropriately labeled. This approach was also taken for the theme “general impact of implantation.” For the *quality of tinnitus* theme, only one subcategory was selected from the initial sample of 37 responses (buzzing), and other subcategories were added based on the commonly reported tinnitus sounds from the literature, for example, “pulsing,” (Henry et al. 2005). Some of these subcategories were not populated. A similar approach was taken for the *provoking factors* theme and for the *impact of tinnitus* theme, where a small number of subcategories were identified for each theme, and further subcategories were added on the basis of previous findings of common reports of provoking factors and the impact of tinnitus (Henry et al. 2005; Tyler et al. 2008). A theme that did not emerge from the initial samples, but was added a priori, was *number of sounds*, as we were interested to determine whether participants would report a complex of multiple tinnitus sounds. This theme was not populated from the responses and was removed. Themes that emerged from responses were those of *location of the tinnitus sound* (divided into subcategories of head, peripheral and other), *immediate impact of the implant*, based on a report of immediate improvement, with subcategories of immediate worsening, and/ or no change noted immediately being added to make the theme exhaustive.

**TABLE 1.**
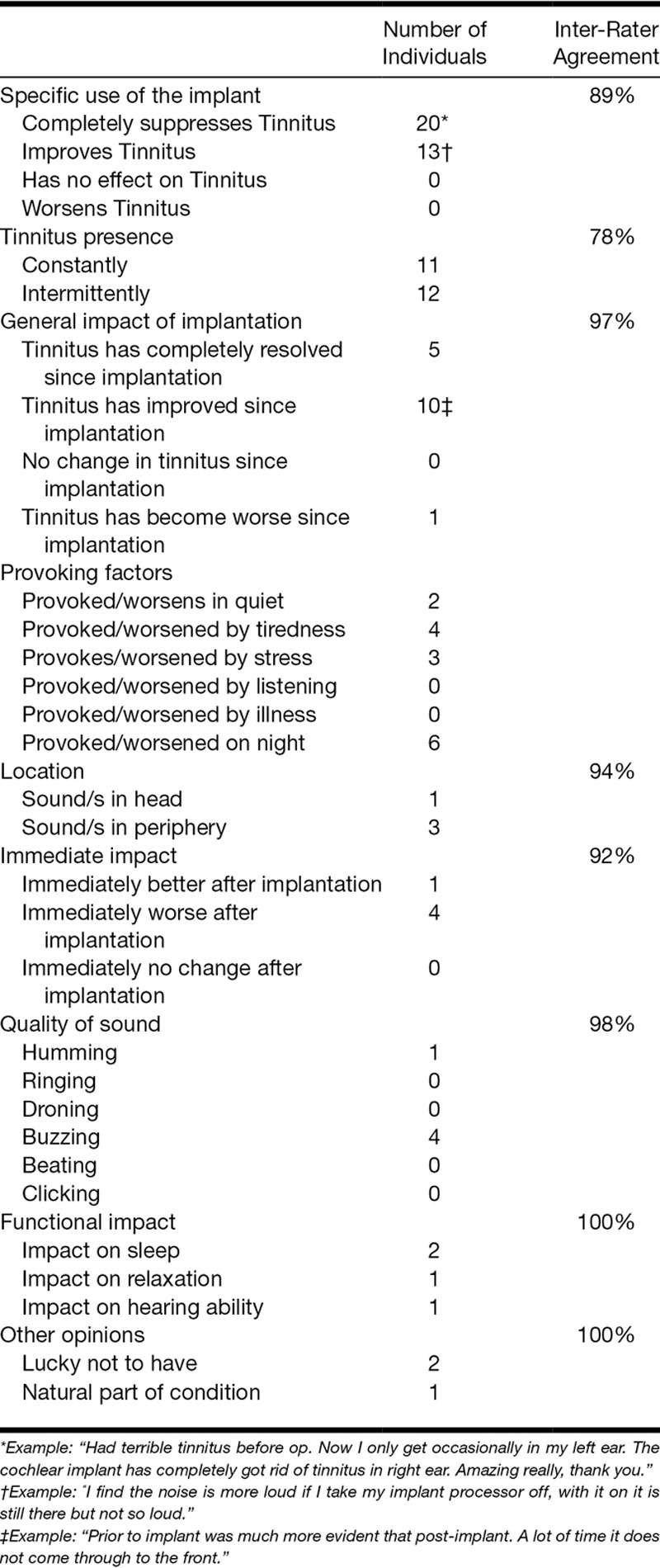
Open text categories and subclassifications

Coding of the initial 37 responses was then independently performed by another experienced clinician (author D.B.). The responses were then analyzed for similarity, with percentage agreement scores determined for each theme. A list of the retained categories and subclassifications are shown in Table 1 along with the inter-rater agreement results, where applicable. It can be seen that the inter-rater agreement was very high, ranging from 78% to 100% across the different categories.

### Ethical Approval

A favorable ethical opinion was granted from the UK Health Research Authority, Research Ethics Committee number: 11/EE/0502.

## RESULTS

### Questionnaire Statistics

It was found that 253 (of 466) questionnaires were returned within an 8-week period (response rate of 54%). Nine of the questionnaires were unusable because the individual had declined consent for use for research purposes or no useable responses were present. Demographic information for those responding to the questionnaire and the full population of adult implant users who received a questionnaire is shown in Table 2.

**TABLE 2.**
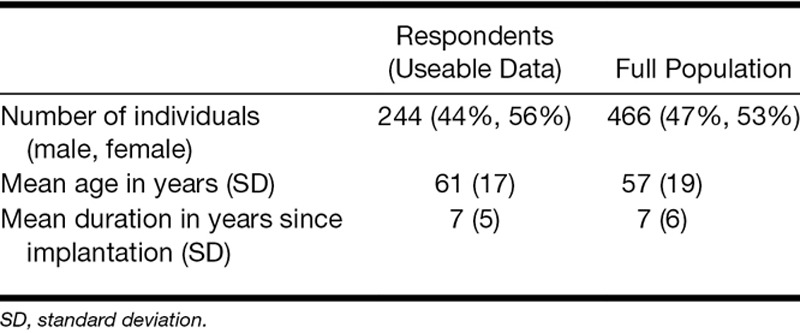
Summary table of demographics of respondents

**Fig. 1.**
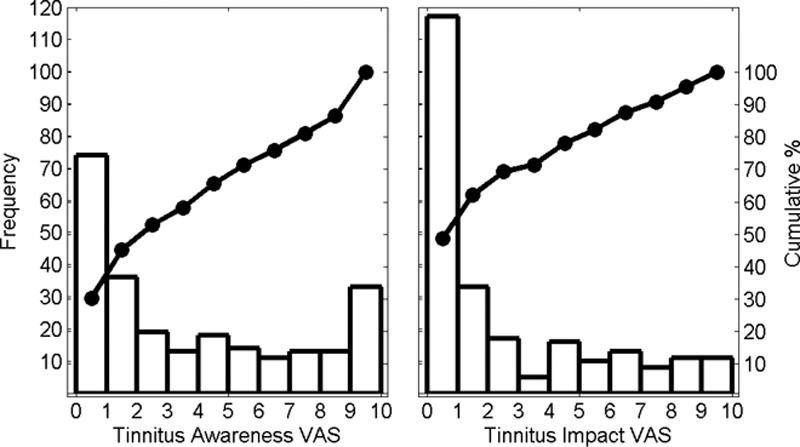
A, Histogram showing the distribution of responses to the tinnitus awareness visual analog scale. B, Histogram showing the distribution of responses to the tinnitus impact visual analog scale. In each case, the leftmost vertical axis shows the frequency of responses and the rightmost vertical axis shows the cumulative value. The filled circles joined by a solid line represent the cumulative distribution.

**Fig. 2.**
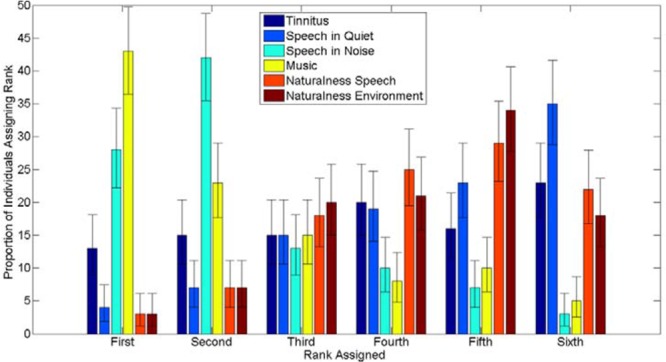
Bar chart showing the distribution of rankings of the separate domains of difficulty for all respondents. The *y* axis indicates the proportion of respondents. The *x* axis indicates the ranking assigned. Different shades indicate different domains of difficulty—see legend for details. The error bars represent 95% confidence intervals calculated using the binomial distribution.

A further 19 respondents left one or more VAS blank. All responses were included in the histograms (shown in Fig. 1) and correlations of tinnitus impact against duration of implantation and age of patient. Data from individuals with one or more VAS response missing were excluded from the ranking and correlation calculations (shown in Fig. 2 and Table 3, respectively).

**TABLE 3.**
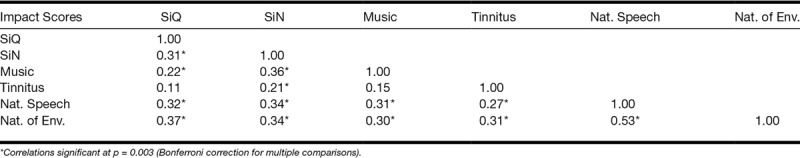
Correlations of the Impact VAS scores across domains

### VAS Scores

Histograms showing the distribution of responses for both *impairment* and *impact for tinnitus* are shown in Figure 1.

**Fig. 3.**
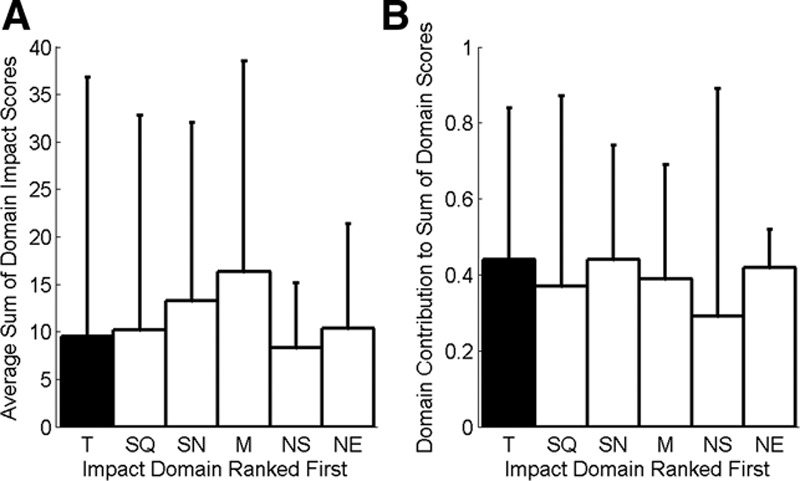
A, Bar chart showing the sum of all *impact* scores across all domains of difficulty when the domain of difficulty as indicated on the *x* axis was ranked first, averaged across individuals. As an example, the filled bar represents the sum of impact scores across domains when the *Tinnitus Impact* domain was ranked first, averaged across individuals. B, Bar chart showing the proportion of the summed *impact* score belonging to the domain of difficulty shown on the *x* axis when that domain was ranked first. As an example, the filled bar represents the relative contribution of the *Tinnitus impact* score to the summed impact scores across all domains when the *Tinnitus Impact* domain was ranked first, averaged across individuals. Nat Env, naturalness of environmental sounds; Nat Sp, naturalness of speech; SiN, speech in noise; SiQ, speech in quiet. Error bars show 95% confidence interval of the distribution.

The histogram for tinnitus awareness (Fig. 1A) shows a wide spread of VAS values. A large proportion of CI users are rarely aware/unaware of tinnitus (74/244, 30%, gave a VAS score of 0 to 1), but 14% (33/244) report tinnitus awareness that is present in the majority/all of the time, with a VAS score greater than nine. There is also a broad distribution of VAS scores for tinnitus impact (Fig. 1B). The proportion of individuals recording a tinnitus VAS score of five or greater is 18%. Eleven individuals (5%) gave a VAS impact score at the most extreme of the range (9 to 10). When the results for the six domains of potential difficulty with the implant were ranked, tinnitus impact was the top-ranking concern for 13% of individuals and ranked first or second for 28% of the CI population who participated (Fig. 2). To assess whether the distribution of ranks varied significantly from chance, a Chi-squared test of independence was calculated. This was significant for the full set of categories (χ^2^ (5, N = 209) →∞, *p* < 0.001) and with *Music* and *Speech in Noise* categories removed (χ^2^ (3, N = 50) =29.5, *p* < 0.001). Similarly, in individuals where tinnitus impact is ranked first, the sum of the impact scores for all domains of difficulty, and the proportion of this score assigned to the tinnitus category, is not significantly different from cases where other difficulties are ranked first (Fig. 3). This suggests that individuals reporting tinnitus as a primary concern find it equally as debilitating as the domains of difficulty ranked first by other individuals.

Correlations between the tinnitus impact scores in each domain are shown in Table 3. Tinnitus impact scores were significantly correlated (when corrected for multiple comparisons) with impact scores for *speech in noise*, *naturalness of speech*, and *naturalness of environmental sounds*. There was no significant correlation between the tinnitus impact VAS score and the duration since implantation (*r* (225) = −0.05, *p* = 0.44) nor the age of the participant at completion of the questionnaire (*r* (225) = −0.04, *p* = 0.59).

Tinnitus awareness and tinnitus impact scores were grouped by CI manufacturer. Significant deviation from normality was observed in histograms and results of statistical tests for both *awareness* and *impact* scores. Kruskal–Wallis (one-way analysis of variance on ranks) showed no significant differences between the mean impact scores (χ =2.917, degree of freedom = 2, *p* = 0.213). No meaningful comparisons between processor types and implant array designs could be drawn due to a relatively large number of different devices present across the population.

Respondents’ clinical logs were assessed for information describing the successful use of the CI. Only one individual was identified as a “relatively poor user,” wearing the device intermittently during the period that questionnaires were completed. Tinnitus awareness and tinnitus impact scores for this individual were both negligible (a value of 0.1 in both cases). For all respondents, sound field hearing thresholds and test results from Bamford–Kowal–Bench (BKB) sentences in quiet and noise (Bench et al. 1979) and Auditory Speech Sound Evaluation 20-phoneme contrasts (Heeren et al. 2012), were obtained, where available, for the map that was in use at the time that the questionnaire was completed. The median percent correct scores were 87% (N = 87), 64% (N = 34), and 95% (N = 168) for BKB-in-quiet, BKB-in-noise, and ASSE 20 phoneme contrasts for the respondent population. Correlations between the three speech measures and tinnitus awareness and tinnitus impact were not significant at the alpha < 0.05 level (two-tailed distribution), although a marginally significant positive correlation was seen between BKB sentence testing in quiet scores and tinnitus awareness (*r* = 0.185, *p* = 0.07). A stepwise linear regression was conducted to investigate the influence of sound field audiometric threshold at each test frequency on tinnitus awareness and tinnitus impact. The sound field threshold at 8 kHz was the only variable to be a significant independent predictor in each case (*F*_awareness_ (1,92) = 7.86, *p* = 0.006; *F*_impact_ (1,90) = 5.86, *p* = 0.018). The correlations were negative (i.e., more acute 8 kHz hearing thresholds were associated with greater tinnitus impact and tinnitus awareness scores), but fairly weak, explaining only 8% and 6% of the variance for tinnitus awareness and tinnitus impact, respectively.

### Open-Text Responses

Open-text responses to the tinnitus question were provided by 107 individuals. The mean tinnitus awareness score was slightly higher for the group who provided written comments than for those that did not (mean = 4.5, SD = 3.4 versus mean = 3.1, SD = 3.3, *p* = 0.004). There was no significant difference for the tinnitus impact scores between the groups providing comments, and the group who did not (mean = 2.8, SD = 3.1 versus mean = 3.1, SD = 3.3, *p* = 0.26). Table 1 summarizes the results of the content analysis and some examples of the respondents’ open-text entries are shown.

## DISCUSSION

This study provides evidence that tinnitus remains a problem for a proportion of adult individuals undergoing cochlear implantation and highlights the need for suitable tinnitus management pathways to be made available alongside typical postimplantation rehabilitation. When the results for the six domains of potential difficulty with the implant were ranked, tinnitus impact was the top ranking concern for 13% of individuals and ranked first or second for 28% of the CI population who participated. Other studies have reported persistent tinnitus awareness in 15 to 50% of the adult-implanted population, although the severity of tinnitus is not commonly described (Tyler & Kelsay 1990; Bredberg et al. 1991; Souliere et al. 1992; Ruckenstein et al. 2001). The data reported here show that these estimates do not necessarily represent individuals in need of tinnitus management since a number of individuals report constant tinnitus awareness but little or no tinnitus distress. The ranking method undertaken may provide a better representation of those individuals in need of tinnitus management because it is less susceptible to external influences that are not directly linked to tinnitus perception. If an absolute estimate is made with the data here, for example, defining a moderate tinnitus impact as being equivalent to a VAS score of five, just under a quarter of the respondents to this question reported a moderate or worse tinnitus severity. A further advantage of using a stealth tinnitus question placed among questions linked to hearing is that non-tinnitus sufferers are included in the population: 30% of individuals with an implant reported essentially no tinnitus awareness (VAS score 0 to 1), and 49% reported no significant impact of tinnitus (VAS score 0 to 1). Clinical logs suggested that the large majority of respondents were good users with fairly high speech discrimination scores. The only significant relationship between subjective measures of performance with the implant and tinnitus was a relatively weak relationship that showed more acute sound field hearing thresholds at 8 kHz were associated with greater tinnitus perception. This is consistent with findings from another study (McKinney et al. 2002), where the population of individuals reporting the most tinnitus distress had the best audiometric thresholds. We also observed a marginal positive correlation between speech discrimination scores and tinnitus awareness, similar to that reported in previous literature (Tyler 1995). At the physiological level, this finding seems to contradict evidence that better electrode positioning, which would be expected to provide better hearing outcomes, is associated with a decreased likelihood of tinnitus perception (Todt et al. 2015). An explanation for this seemingly counterintuitive finding is that the presence of tinnitus may have influenced candidacy for implantation and the choice of ear to receive the device within this population. This could result in a bias toward marginally better auditory function in the tinnitus sufferers undergoing implantation compared to the non-tinnitus sufferers. Further research is required to clarify the causative relationship between differences in the auditory physiology and electrode placement between individual CI users and the subsequent impact on auditory outcomes and tinnitus.

A limitation of this cross-sectional study is the risk of participation bias. While our response rate (54%) is comparable to other studies, because it is less than 100%, respondents self-select their participation, and this may have introduced some bias. Table 2 demonstrates that the respondent population shows similar demographics to the overall implant population who were sent questionnaires, with the exception that the respondents were, on average, older than the nonrespondents. This may reflect the fact that retired individuals may have had more time to complete and return the questionnaire. In the context of tinnitus, the impact of this difference between the responding/nonresponding groups is not readily obvious. One indicator that this age difference may not introduce a large bias is that fact that we did not see a relationship between an individual’s age and tinnitus scores, albeit within the respondent population.

Open text responses suggest that, at least for some individuals, the benefits obtained from the implant are primarily due to masking, or linked to acute effects of electrical stimulation, rather than a more permanent alteration due to plasticity. The role of masking as the primary mechanism of benefit is also supported by the lack of correlation between the duration since implantation and tinnitus impact. The most common open-text responses were to report an improvement with specific CI use, either complete resolution or some improvement (see Table 1). In some cases, the improvement with use was communicated as a description that the tinnitus was worse/present only with the implant off. The reports that tinnitus was worse during the night (six individuals) could also reflect the same increased awareness of tinnitus due to reduced external sound stimulation (either because the environment is quieter or because the implant is removed). Sound enrichment is a common management approach used to alleviate tinnitus distress at such times for tinnitus sufferers with acoustic hearing (Hobson et al. 2010), and these findings highlight a need to consider similar approaches to tinnitus management in CI users, that go beyond alterations to processor settings, but may include changes in patterns of use and the use of external stimuli.

One individual reported that they were aware that the tinnitus became worse immediately after the implantation procedure, but that it had resolved since this time. There were no other reports of tinnitus becoming worse since implantation. While the absence of such comments cannot confirm that this was true for all individuals, there is no evidence in the results presented here that would contradict the notion that it is rare for tinnitus awareness and distress to increase as a result of implantation. Similar to the reported results from studies that have looked at the characteristics of tinnitus in the wider population, other open-text responses show a significant degree of heterogeneity of tinnitus experiences, for example, in the wide variety of descriptors used.

The relatively large population studied here comprised individuals with a number of different devices. There were no statistical differences in the mean tinnitus impact score between implant manufacturers. This is perhaps unsurprising as the design of implant processors and electrodes has not yet focused on tinnitus alleviation as a primary goal. A prospective study examining the influence of electrode insertion depth and configuration on tinnitus perception would test the hypothesis that cochlear place and stimulation rate parameters may be important for tinnitus suppression (Zeng et al. 2011).

No clear trend for tinnitus to decrease with time since implantation was observed, and most open-text responses described benefit specific to the use of the device rather than sustained relief when the device is removed. The findings suggest that masking rather than reversal of maladaptive plasticity is more likely to be the primary mechanism of tinnitus alleviation/improvement in this population.

The fact that the implant does not replicate normal peripheral auditory function might be responsible for limitations in tinnitus alleviation. The information available to the listener is usually dominated by envelope cues (slower modulations), and, even when processing strategies that attempt to introduce “temporal fine structure” information, the ability of CI users to decode this information is severely limited (Carlyon et al. 2010; Zeng et al. 2014). Some processing strategies are deliberately sparse to minimize the impact of current spread from an electrode reaching nerve populations. In all cases, there are fewer, and broader, auditory channels available. The implant can only stimulate nerve fibers that are present and functioning, and there is variability in populations of surviving spiral ganglia, peripheral processes, and inner hair cells across individuals (Kujawa & Liberman 2015). Consequently, there is a fundamental restriction of information reaching the auditory cortex even in the case of a highly successful implantation procedure. All of these factors may impact the ability of the implant to reverse “maladaptive plasticity” purportedly associated with tinnitus (Meredith et al. 2012). Additionally, there are features of CI use that may be less than optimal for tinnitus perception. In the United Kingdom, the majority of adult implant users have a single device, and monaural stimulation of the auditory system provides the obvious limitation that peripheral masking of the tinnitus signal is restricted to the implanted ear. The significant asymmetry in input to the auditory cortex is regarded, in itself, as a potential trigger for tinnitus according to the popular neurophysiological model (Jastreboff & Hazell 1993), although the low number of cases of implant-induced tinnitus cast doubt on this assertion (Baguley & Atlas 2007). A further issue is the extended period of time without sound input when the processor is removed at night during which tinnitus distress may be significant, and a negative impact on the ability to sleep as a result of tinnitus is common in the tinnitus suffering population (Axelsson & Ringdahl 1989; Hallam 1996; Khedr et al. 2010). The consequence of this research is that clinicians, scientists, and CI manufacturers should consider that tinnitus awareness is persistent and problematic for many individuals, and the impact may be more important than the limitations faced due to impaired auditory performance in some situations. Clinical and research activities and funding should reflect this need.

## ACKNOWLEDGMENTS

The authors thank the following: All participants for their time spent completing the questionnaires; Eldre Beukes, Frances Harris, Susan Fields, and Tracy Church for assistance in accessing patient databases, Karen Shammas for processing of data, and Alison MacLeod for providing comments on the questionnaire design.

## Supplementary Material
