# Supplementary material for: A Cross-Sectional Questionnaire Study of Tinnitus Awareness and Impact in a Population of Adult Cochlear Implant Users
Source: Ear Hear. 2018 Dec 28;40(1):135–42. doi: 10.1097/AUD.0000000000000601 (PMC6319580; doi:10.1097/AUD.0000000000000601)
Supplement: Supplementary file 1 [file aud-40-135-s001.pdf]

Thank you for sparing some time to complete this questionnaire. Please read these instructions before completing.

There are six separate questions. In each case you will be required to make a mark on a line to indicate your answer. Carefully draw a cross in black ink at a point along the line which you feel describes your own experience.

Below is an example question with a typical answer. This is not related to hearing and is included as a guide:

EXAMPLE:

How difficult is it for you to use a computer?

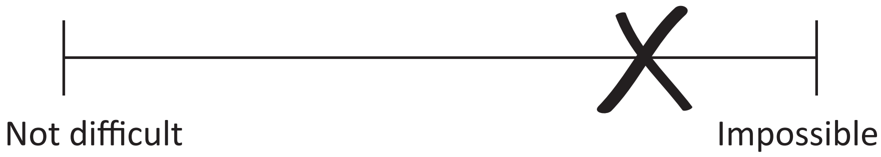

If you would like to add any comments about each question there is space for you to do so. You do not have to make comments if you do not want to.

If you have any queries please contact Phil Gomersall using the following:

Email: philip.gomersall@mrc-cbsu.cam.ac.uk
Tel: 01223 217797
Post: Phil Gomersall, Audiology Department Clinic 10, Addenbrooke's Hospital, Hills Road Cambridge, CB2 0QQ

Once you have completed the questionnaire please return it in the envelope provided. The postage has already been paid.

Please note: THIS QUESTIONNAIRE IS PRINTED ON BOTH SIDES OF THE PAPER

Question 1
How difficult is it for you to understand a single person speaking in a quiet room?

Not difficult — Impossible

How much does this upset/annoy/worry you?

Not at all — Very much

Comments..
....
....
....

Question 2
How difficult is it for you to understand a single person speaking in background noise?

Not difficult — Impossible

How much does this upset/annoy/worry you?

Not at all — Very much

Comments..
....
....
....

Question 3

How difficult is it for you to follow music?

Not difficult — Impossible

How much does this upset/annoy/worry you?

Not at all — Very much

Comments..
....
....
....

Question 4

How often are you aware of any buzzing, ringing or other noises in your head or ears (tinnitus)?

Never — All of the time

How much does this upset/annoy/worry you?

Not at all — Very much

Comments..
....
....
....

Question 5

How natural are people's voices?

Completely natural Very unnatural

How much does this upset/annoy/worry you?

Not at all Very much

Comments...
....
....
....

Question 6

How natural are everyday sounds other than people talking (for example running water)?

Completely natural Very unnatural

How much does this upset/annoy/worry you?

Not at all Very much

Comments...
....
....
....

END OF QUESTIONNAIRE
